# Gastrointestinal involvement in a patient with familial Mediterranean fever mimicking Crohn’s disease: a case report

**DOI:** 10.1007/s12328-021-01426-2

**Published:** 2021-05-11

**Authors:** Yoshihiro Yokoyama, Tsukasa Yamakawa, Tadashi Ichimiya, Tomoe Kazama, Daisuke Hirayama, Kohei Wagatsuma, Hiroshi Nakase

**Affiliations:** Department of Gastroenterology and Hepatology, Sapporo Medical University School of Medicine, Chuo-ku, Sapporo, Hokkaido 060-8543 Japan

**Keywords:** Familial Mediterranean fever, *MEFV*, Crohn’s disease, Inflammatory bowel disease unclassified

## Abstract

Familial Mediterranean fever (FMF) in gastrointestinal involvement has been considered rare, but resent reports suggest that FMF causes enterocolitis which is similar endoscopic findings to inflammatory bowel disease. The clinical characteristics and endoscopic findings of FMF with enterocolitis remain unclear. Here, we report a case of an FMF patient who had enterocolitis with stricture of the terminal ileum whose endoscopic and clinical features mimicked Crohn’s disease. A 23-year-old man who was diagnosed with FMF 10 years ago presented with abdominal pain and diarrhea. Colonoscopy showed terminal ileitis and aphthous colitis; however, these findings, including the histopathology, did not confirm Crohn’s disease. Therefore, we diagnosed FMF with enterocolitis and administered anti-interleukin-1β monoclonal antibody (canakinumab). The patient’s symptoms improved with treatment, but after 1 year, lower abdominal pain recurred. Colonoscopy revealed a stricture of the terminal ileum. Endoscopic balloon dilation relieved his symptoms. At present, he has been followed up without surgical treatment by endoscopic balloon dilation every 6 month. Clinicians should be aware that FMF accompanied with enterocolitis may resemble Crohn’s disease.

## Introduction

Inflammatory bowel disease (IBD) is a chronic inflammatory disease of the intestinal tract that includes ulcerative colitis (UC) and Crohn's disease (CD). The typical endoscopic findings are diffuse mucosal inflammation continuous from the rectum in UC, and longitudinal ulcers and a cobble stone appearance in CD. However, there are cases that are difficult to classify as UC or CD, which are called inflammatory bowel disease unclassified (IBDU) in Japan. In 2012, we first reported an IBDU patient with a heterozygous G304R mutation in the *MEFV* gene, who drastically responded to colchicine treatment alone [1]. Following this report, there have been several reports regarding IBDU patients with *MEFV* mutations responding to colchicine [2–6]. Based on these clinical cases, we advocated the existence of “*MEFV* gene-related enterocolitis” within the classification of IBDU. In addition, it has been clarified that familial Mediterranean fever (FMF) causes not only abdominal attacks due to serositis, but also gastrointestinal involvement, which is similar to endoscopic findings in IBD [7–11]. The clinical characteristics of *MEFV* gene-related enterocolitis, including endoscopic findings, remain unclear despite the increasing number of reported cases. We report an FMF patient who had enterocolitis with stricture of the terminal ileum whose endoscopic and clinical features mimicked CD.

## Case report

**Fig. 1:**
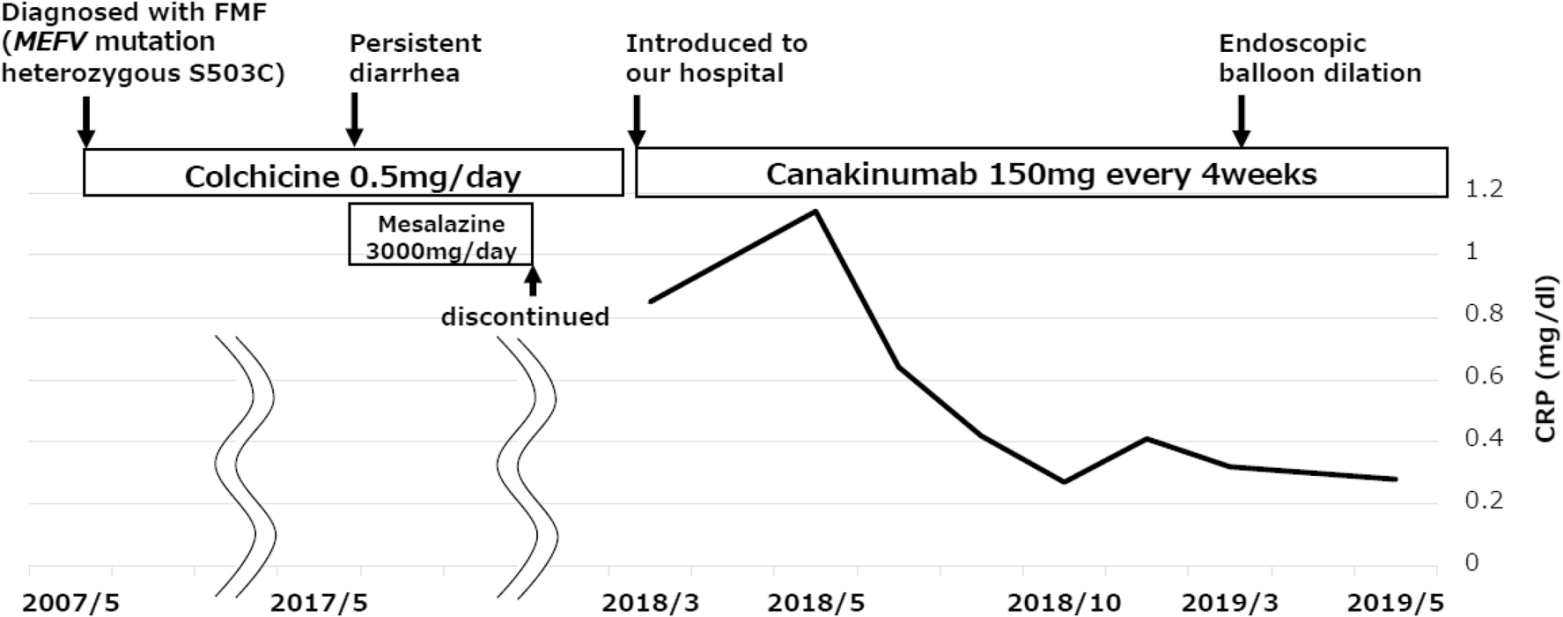
Clinical course of this patient. C-reactive protein (CRP) has improved after the administration of canakinumab

A 23-year-old Japanese man visited our hospital in March 2018 march because of abdominal pain, diarrhea, and joint pain. He had a history of intermittent fever over 38 °C with elevation of C-reactive protein (CRP) and had been diagnosed with interstitial nephritis in January 2006. Moreover, he experienced abdominal pain and joint pain with intermittent fever lasting more than 2 months in May 2007. Based on his clinical symptoms and a heterozygous S503C mutation in the *MEFV* gene, he was diagnosed with FMF (Fig. 1). The administration of colchicine improved his symptoms, but he had diarrhea with increasing dosage. Therefore, he had been treated with colchicine 0.5 mg/day and was stable without FMF relapse for about 10 years after starting colchicine.

**Fig. 2:**
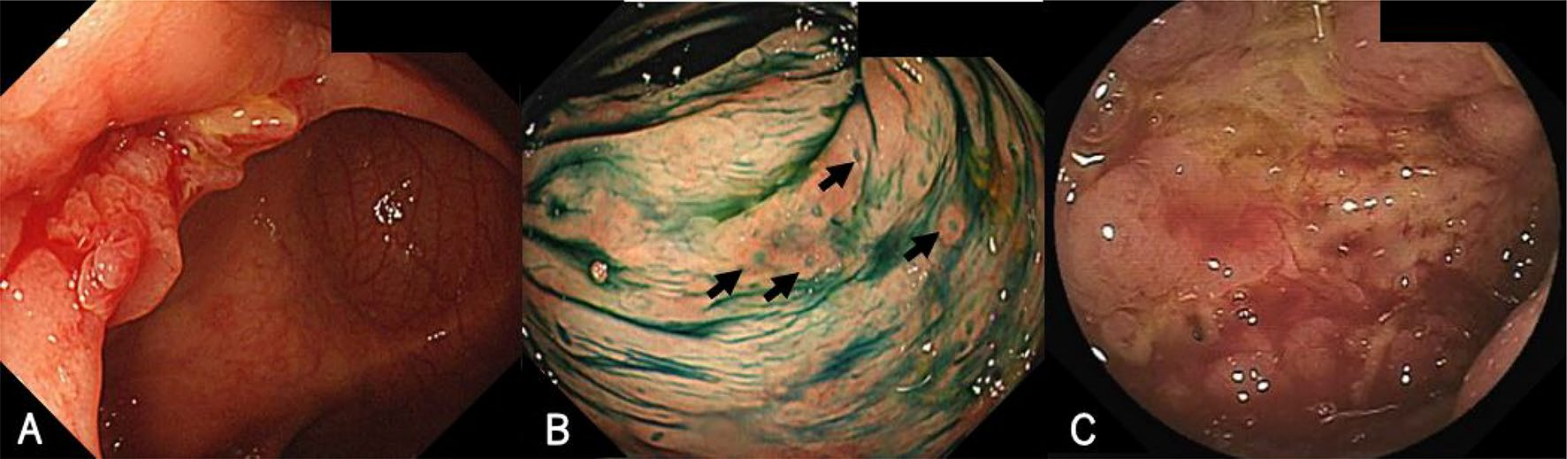
Colonoscopic findings show edema and erosion in the ileocecal valve (**a**) and aphthous colitis (arrows) in the sigmoid colon (**b**). Double balloon endoscopy shows ulceration in the terminal ileum (**c**)

In May 2017, he complained of persistent diarrhea for 2 months, and colonoscopy revealed edema and erosion in the ileocecal valve and aphthous lesions in the cecum and sigmoid colon (Fig. 2a, b). Moreover, a double-balloon endoscopy showed ulceration with mild stricture in the terminal ileum (Fig. 2c). Histology of the biopsy did not reveal granuloma or amyloid deposition. His clinical data, including endoscopic findings, did not meet the diagnostic criteria for CD. Therefore, he was diagnosed with IBDU and received mesalazine 3000 mg/day. His symptoms gradually improved after treatment, and mesalazine was discontinued.

In March 2018, he was admitted to our hospital because of recurrent diarrhea, abdominal pain, intermittent fever, and joint pain. He did not take any non-steroidal anti-inflammatory drugs (NSAIDs). Laboratory data showed a slight elevation of CRP and serum amyloid A (SAA) (Table 1). The T-SPOT.TB test, which is one of the interferon-gamma releasing assays for tuberculosis was negative. Based on his history of FMF, we diagnosed him with FMF accompanied with enterocolitis. Because he was refractory to colchicine, we decided to administer anti-interleukin (IL)-1β monoclonal antibody, canakinumab (ILARIS®, Novartis Pharmaceuticals Corporation, Basel, Switzerland). His joint pain and diarrhea subsided, and CRP and SAA gradually decreased with the administration of 150 mg by subcutaneous injection every 4 weeks.

**Table 1:** Laboratory data on admission

WBC	5800/μL	TP	6.9 g/dL	Na	141 mEq/L
Neu	4150/μL	Alb	4.3 g/dL	Cl	105 mEq/L
Lym	1030/μL	T-bil	0.8 IU/L	K	3.6 mEq/L
RBC	4.73 × 10^6^/μL	AST	31 IU/L	CRP	0.85 mg/dL
Hb	14.2 g/dL	ALT	75 IU/L	SAA	21.5 μg/mL
Hct	42.6%	ALP	352 IU/L	T-SPOT.TB	(−)
Plt	369 × 10^3^/μL	BUN	12 mg/dL	CMV IgG	(+)
		Cr	0.82 mg/dL		

WBC: white blood cell, Neu: neutrophils, Lym: lymphocytes, RBC: red blood cell, Hb: hemoglobin, Hct: hematocrit, Plt: platelets, TP: total protein, Alb: albumin, T-bil: total bilirubin, AST: aspartate aminotransferase, ALT: alanine aminotransferase, ALP: alkaline phosphatase, BUN: blood urea nitrogen, Cr: creatinine, Na: serum sodium, Cl: serum chloride, K: serum potassium, CRP: C-reactive protein, SAA: serum amyloid A, CMV: cytomegalovirus, IgG: immunoglobulin G

**Fig. 3:**
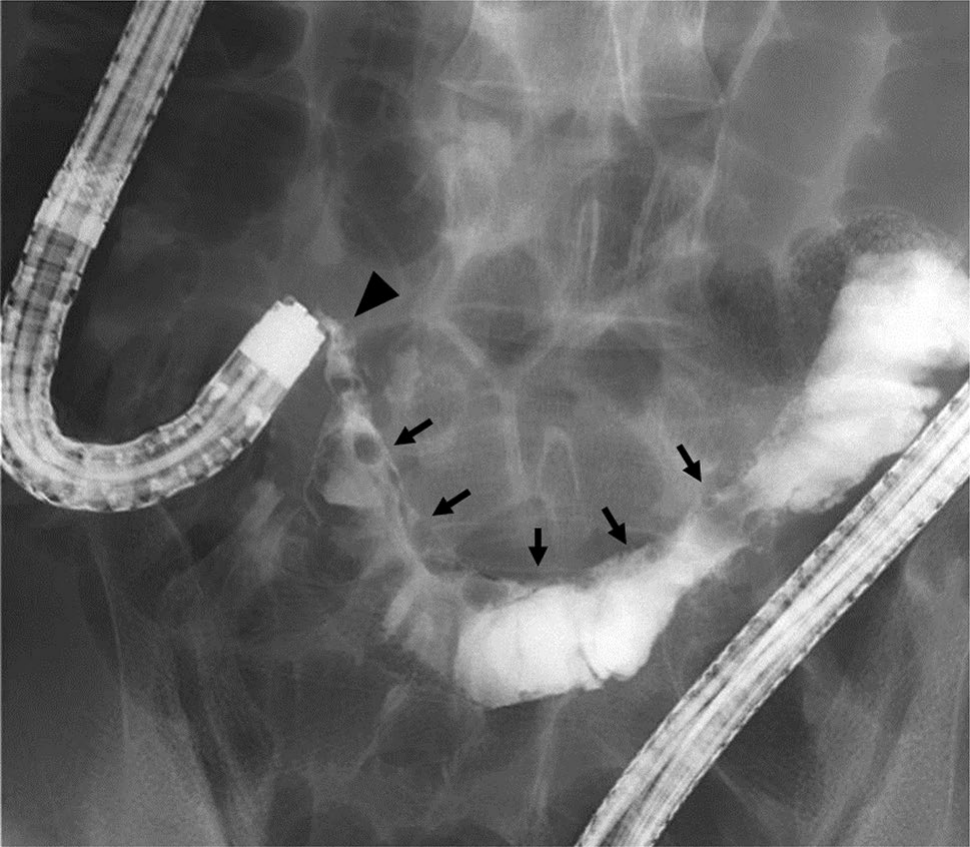
Fluoroscopy shows long segmental stiff appearance at the mesenteric side (arrows) and stricture (arrow head) in the terminal ileum

**Fig. 4:**
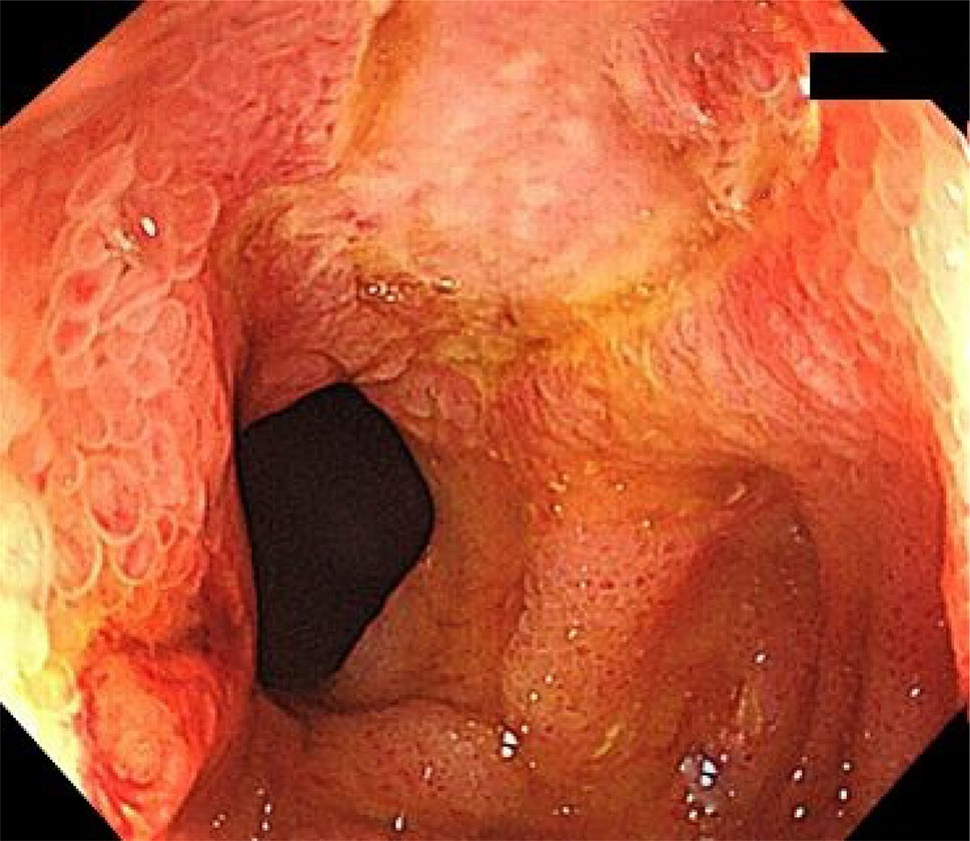
Colonoscopic findings during endoscopic balloon dilation show round ulcers and scars in the terminal ileum

However, he repeatedly complained of postprandial nausea and lower abdominal pain during treatment with canakinumab. We performed colonoscopy and the scope did not pass through the terminal ileum due to stricture. Fluoroscopy showed a long segmental stiff appearance on the mesenteric side of the terminal ileum (Fig. 3). We performed endoscopic balloon dilatation (EBD) with a 10–12 mm diameter balloon (CRE™ Wire guided balloon dilatation catheter, Boston Scientific, Marlborough, MA, USA) for the terminal ileal stricture. Colonoscopic findings showed round ulcers and scars in the terminal ileum (Fig. 4). After the EBD, the patient’s abdominal symptoms improved. At present, he has received routine EBD every 6 months to avoid ileal stricture.

## Discussion

We herein report an FMF patient who had enterocolitis mimicking CD. Canakinumab administration relieved his FMF symptoms, such as fever and joint pain. However, abdominal pain due to stricture of the terminal ileum appeared during canakinumab treatment. Repeated EBD improved his abdominal symptoms and avoided surgical intervention.

FMF is a hereditary autoinflammatory disease that is characterized by periodic fever and serositis. *MEFV* was identified in 1997 as the gene responsible for FMF [12]. *The MEFV* gene is composed of 10 exons, and codes for the pyrin protein. Pyrin regulates caspase-1 activation and suppresses the activation of IL-1β in the inflammasomes. It is considered that functional abnormality of the pyrin protein causes FMF [13, 14].

The Tel-Hashomer criteria published in 1997 are often used for the diagnosis of FMF [15]. FMF involvement in gastrointestinal lesions has been considered rare, but recent reports have shown gastrointestinal involvement of FMF in the colon as well as in the small intestine, including the duodenum [4, 5, 7]. However, the endoscopic features of FMF with enterocolitis have not yet been clarified. Based on previous reports, pseudopolyposis-like lesions and right-sided UC-like mucosal lesions with rectal sparing might be the features of endoscopic findings of FMF with enterocolitis [1, 6]. In small intestine lesions, several endoscopic findings such as redness, aphtha, erosion, and ulcer have been reported [7, 11]. Furthermore, there are some reports of FMF patients with terminal ileum lesions mimicking Crohn's disease [3, 4, 8]. However, there have been no reports on the association of the site of genetic mutation with the morphology of gastrointestinal lesions. In our case, fluoroscopy showed a long segmental stiff appearance on the mesenteric side, which looked like a longitudinal ulcer in the terminal ileum, while endoscopic findings showed only round ulcers and scars without longitudinal ulcers. To explain the discrepancy between the fluoroscopic and endoscopic findings, we considered that fluoroscopy could reflect the serositis caused by FMF on the mesenteric side of the terminal ileum. The longitudinal ulcer-like appearance on the mesenteric side might have confused the diagnosis if the patient had not initially complained of FMF symptoms and received analysis of the *MEFV* gene. Therefore, physicians should not quickly diagnose patients with enterocolitis as UC or CD only based on endoscopic findings. A comprehensive diagnosis requires careful attention to the patient’s symptoms and a general examination.

The first-line FMF treatment is colchicine, which is reported to be effective in almost 90% of cases [16, 17]. In *MEFV* gene-related enterocolitis, colchicine is also effective. Recently, the usefulness of canakinumab for colchicine-resistant FMF cases has been reported [18]. Canakinumab is an antibody that suppresses the overexpression of IL-1β. Moreover, Takahashi et al. reported the effectiveness of anti-TNF-α antibody for colchicine-resistant FMF patients with enterocolitis [8]. We selected canakinumab rather than anti-TNF-α antibody because the pathogenesis of FMF is associated with overexpression of IL-1β. This is the first reported case of colchicine-resistant FMF with enterocolitis who received canakinumab. He had an improvement in clinical symptoms and a decrease in inflammatory biomarkers such as CRP and SAA after starting canakinumab. However, the stricture of the terminal ileum appeared during the healing process. EBD was effective for the stricture, and he has been able to avoid surgical treatment by repeated EBD. However, whether anti-TNF-α antibody could relieve his symptoms and prevent the stricture of the terminal ileum remains unclear.

In conclusion, this case strongly suggests that FMF patients may have accompanied enterocolitis, which could have a clinical course similar to that of CD. Further cases are required to elucidate the pathogenesis of FMF with enterocolitis.

## Abbreviations

IBD: Inflammatory bowel disease
UC: Ulcerative colitis
CD: Crohn’s disease
IBDU: Inflammatory bowel disease unclassified
FMF: Familial Mediterranean fever
CRP: C-reactive protein
SAA: Serum amyloid A
IL: Interleukin
EBD: Endoscopic balloon dilation

## References

[1] Arasawa S, Nakase H, Ozaki Y (2012). Mediterranean mimicker. Lancet.

[2] Matsumoto S, Urayoshi S, Yoshida Y (2014). Familial Mediterranean fever in which Crohn’s disease was suspected: a case report. BMC Res Notes.

[3] Asakura K, Yanai S, Nakamura S (2018). Familial Mediterranean fever mimicking Crohn disease: a case report. Medicine (Baltimore).

[4] Torisu T, Kawatoko S, Esaki M (2017). Febrile attacks with a refractory colonic lesion. Gastroenterology.

[5] Esaki M, Kawano S, Matsumoto T (2017). Rare cause of duodenojejunal pseudopolyposis: report of a case of adult-onset familial Mediterranean fever. Dis Endosc.

[6] Saito D, Hibi N, Ozaki R (2020). MEFV gene-related enterocolitis account for some cases diagnosed as inflammatory bowel disease unclassified. Digestion.

[7] Kitade T, Horiki N, Katsurahara M (2015). Usefulness of small intestinal endoscopy in a case of adult-onset familial mediterranean fever associated with Jejunoileitis. Intern Med.

[8] Takahashi T, Tsukuda H, Itoh H (2012). An atypical familial Mediterranean fever patient who developed ulcers in the terminal ileum and recurrent abscess-like lesions in multiple organs. Intern Med.

[9] Yorifuji N, Kakimoto K, Higuchi K (2015). Recurrent abdominal pain accompanied by small intestinal lesions. Gastroenterology.

[10] Agin M, Tumgor G, Kont A (2018). Endoscopic findings in patients with familial Mediterranean fever and dyspeptic symptoms. Prz Gastroenterol.

[11] Demir A, Akyüz F, Göktürk S (2014). Small bowel mucosal damage in familial Mediterranean fever: results of capsule endoscopy screening. Scand J Gastroenterol.

[12] French FMF Consortium (1997). A candidate gene for familial Mediterranean fever. Nat Genet.

[13] Özen S, Batu ED, Demir S (2017). Familial Mediterranean fever: recent developments in pathogenesis and new recommendations for management. Front Immunol.

[14] Onen F (2006). Familial Mediterranean fever. Rheumatol Int.

[15] Livneh A, Langevitz P, Zemer D (1997). Criteria for the diagnosis of familial Mediterranean fever. Arthritis Rheum.

[16] Migita K, Uehara R, Nakamura Y (2012). Familial Mediterranean fever in Japan. Medicine (Baltimore).

[17] Alghamdi M (2017). Familial Mediterranean fever, review of the literature. Clin Rheumatol.

[18] Berdeli A, Şenol Ö, Talay G (2019). Treatment of familial mediterranean fever with canakinumab in patients who are unresponsive to colchicine. Eur J Rheumatol.

